# Spanish Pediatric Inflammatory Bowel Disease Diagnostic Delay Registry: SPIDER Study From Sociedad Española de Gastroenterología, Hepatología y Nutrición Pediátrica

**DOI:** 10.3389/fped.2020.584278

**Published:** 2020-10-15

**Authors:** Santiago Jiménez Treviño, Gemma Pujol Muncunill, Rafael Martín-Masot, Alejandro Rodríguez Martínez, Oscar Segarra Cantón, Luis Peña Quintana, Honorio Armas Ramos, Francisco Javier Eizaguirre Arocena, Josefa Barrio Torres, José Ignacio García Burriel, Luis Ortigosa Castillo, Ester Donat Aliaga, Vanesa Crujeiras Martínez, Patricia Barros García, Gonzalo Botija Arcos, Juan Manuel Bartolomé Porro, Mercedes Juste Ruiz, Carlos Ochoa Sangrador, Zuriñe García Casales, Gonzalo Galicia Poblet, Pablo Oliver Goicolea, Helena Lorenzo Garrido, Ruth García Romero, Enrique La Orden Izquierdo, David Pérez Solis, Víctor Manuel Navas-López, Juan José Díaz Martin, Javier Martín de Carpi

**Affiliations:** ^1^Pediatrics Oviedo, Hospital Universitario Central de Asturias, Asturias, Spain; ^2^Unit for the Comprehensive Care of Paediatric Inflammatory Bowel Disease, Hospital Sant Joan de Deu, Barcelona, Spain; ^3^Pediatric Gastroenterology and Nutrition Unit, Hospital Regional Universitario de Málaga, Málaga, Spain; ^4^Pediatric Gastroenterology and Nutrition Unit, Hospital Universitario Virgen del Rocío, Sevilla, Spain; ^5^Pediatric Gastroenterology and Nutrition Unit, Hospital Vall d'Hebrón, Barcelona, Spain; ^6^Pediatric Gastroenterology and Nutrition Unit, Hospital Universitario Materno Infantil de Canarias, Las Palmas Gran Canaria, Spain; ^7^Pediatric Gastroenterology and Nutrition Unit, Hospital Universitario de Canarias, La Laguna, Spain; ^8^Pediatric Gastroenterology and Nutrition Unit, Hospital Universitario de Donostia, San Sebastián, Spain; ^9^Pediatric Gastroenterology and Nutrition Unit, Hospital Universitario de Fuenlabrada, Fuenlabrada, Spain; ^10^Pediatric Gastroenterology and Nutrition Unit, Complexo Hospitalario Universitario de Vigo, Vigo, Spain; ^11^Pediatric Gastroenterology and Nutrition Unit, Hospital Universitario Nuestra Señora de la Candelaria, Santa Cruz de Tenerife, Spain; ^12^Pediatric Gastroenterology and Nutrition Unit, Hospital La Fe, Valencia, Spain; ^13^Pediatric Gastroenterology and Nutrition Unit, University Hospital of Santiago de Compostela, Santiago de Compostela, Spain; ^14^Pediatric Gastroenterology and Nutrition Unit, Hospital San Pedro de Alcántara, Cáceres, Spain; ^15^Pediatric Gastroenterology and Nutrition Unit, San Rafael Hospital, Madrid, Spain; ^16^Pediatric Gastroenterology and Nutrition Unit, Hospital Rio Carrion, Palencia, Spain; ^17^Pediatric Gastroenterology and Nutrition Unit, Hospital Universitari San Juan de Alicante, Sant Joan d'Alacant, Spain; ^18^Pediatric Gastroenterology and Nutrition Unit, Hospital Virgen de la Concha, Zamora, Spain; ^19^Pediatric Gastroenterology and Nutrition Unit, Hospital Universitario Araba Sede Txagorritxu, Vitoria-Gasteiz, Spain; ^20^Pediatric Gastroenterology and Nutrition Unit, Hospital General Universitario de Guadalajara, Guadalajara, Spain; ^21^Pediatric Gastroenterology and Nutrition Unit, Hospital de Mendaro, Mendaro, Spain; ^22^Pediatric Gastroenterology and Nutrition Unit, Hospital de Basurto, Basurto, Spain; ^23^Pediatric Gastroenterology and Nutrition Unit, Hospital Universitario Miguel Servet, Zaragoza, Spain; ^24^Pediatric Gastroenterology and Nutrition Unit, Hospital Universitario Infanta Elena, Valdemoro, Spain; ^25^Pediatric Gastroenterology and Nutrition Unit, Hospital San Agustín de Avilés, Avilés, Spain

**Keywords:** inflammatory bowel disease, Crohn's disease, ulcerative colitis, diagnostic delay, time to diagnosis, children

## Abstract

**Background and Aims:** Diagnostic delay (DD) is especially relevant in children with inflammatory bowel disease, leading to potential complications. We examined the intervals and factors for DD in the pediatric population of Spain.

**Methods:** We conducted a multicentric prospective study, including 149 pediatric inflammatory bowel disease patients, obtaining clinical, anthropometric, and biochemical data. Time to diagnosis (TD) was divided into several intervals to identify those where the DD was longer and find the variables that prolonged those intervals. Missed opportunities for diagnosis (MODs) were also identified.

**Results:** Overall TD was 4.4 months (interquartile range [IQR] 2.6–10.4), being significantly higher in Crohn's disease (CD) than in ulcerative colitis (UC) (6.3 [IQR 3.3–12.3] vs. 3 [IQR 1.6–5.6] months, p = 0.0001). Time from the visit to the first physician until referral to a pediatric gastroenterologist was the main contributor to TD (2.4 months [IQR 1.03–7.17] in CD vs. 0.83 months [IQR 0.30–2.50] in UC, p = 0.0001). One hundred and ten patients (78.3%) visited more than one physician (29.9% to 4 or more), and 16.3% visited the same physician more than six times before being assessed by the pediatric gastroenterologist. The number of MODs was significantly higher in CD than that in UC patients: 4 MODs (IQR 2–7) vs. 2 MODs ([IQR 1–5], p = 0.003). Referral by pediatricians from hospital care allowed earlier IBD diagnosis (odds ratio 3.2 [95% confidence interval 1.1–8.9], p = 0.025).

**Conclusions:** TD and DD were significantly higher in CD than those in UC. IBD patients (especially those with CD) undergo a large number of medical visits until the final diagnosis.

## Introduction

Pediatric inflammatory bowel disease (PIBD) groups together a number of chronic disorders with poorly understood etiology, including Crohn's disease (CD), ulcerative colitis (UC), and inflammatory bowel disease unclassified (IBD-U) (1). Although its incidence has increased in recent years (2), PIBD remains infrequent in primary care clinics (3). Primary care pediatricians (PCPs) visit patients with diseases that display IBD symptoms but with a much higher prevalence (functional gastrointestinal disorders, infectious gastroenteritis, celiac disease, etc.), which it should be noted, account for a high number of visits they must face daily. These three factors, low prevalence of PIBD, attention to more prevalent diseases, and heavy caseloads, might condition a lack of diagnostic suspicion and, therefore, a delay in the diagnosis of inflammatory bowel disease (IBD) (4).

Time to diagnosis (TD) is defined as the time interval from the patient's onset of symptoms to the final diagnosis of IBD (5) and is especially relevant in children. Missing school days and social isolation secondary to the disease and the undue prolongation of the diagnostic process are some of the potential consequences of prolonged TD (4). Diagnosis delay (DD) in PIBD is associated with an increased risk of complications (6, 7), growth failure and delayed puberty (8), more extensive disease (3, 9, 10), worse response to medical treatment (9), greater need for surgery (6), and lower health-related quality of life (11). The objectives of the present study were to describe the total duration of the PIBD diagnosis in Spain and the duration of the different subintervals that could build total TD and to identify factors associated with DD.

## Patients and Methods

We conducted a multicentric prospective cohort study that included pediatric patients diagnosed with IBD between 2014 and 2015, based on clinical, laboratory, endoscopic, radiological, and histological criteria, according to European Society for Pediatric Gastroenterology Revised Porto Criteria for the diagnosis of IBD in children and adolescents (12). The participating centers were invited through the distribution list of the Spanish Society of Pediatric Gastroenterology, Hepatology, and Nutrition (Sociedad Española de Gastroenterología, Hepatología y Nutrición Pediátrica). A case report form (CRF) was designed and distributed by email to the participating centers. Written informed consent was obtained from parents and also from patients older than 12 years old before the inclusion in the study and before collecting the data on the CRF. The CRF was to be completed and sent to the coordinator within 1 month from diagnosis.

TD was defined as the time between symptom onset and IBD diagnosis. The following subintervals were also defined: Interval 1: time from the onset of symptoms to the first consultation with a physician; Interval 2: time from initial physician's visit until IBD diagnosis. Interval 2 was divided into three subintervals: Interval 2a, time from the first physician's visit until referral to a pediatric gastroenterologist (PG); Interval 2b: time from referral to the PG until the PG office visit; Interval 2c: time from the PG visit until IBD diagnosis.

DD was defined as an overall TD greater than the upper quartile. Subintervals were similarly defined as prolonged if they exceeded the upper quartile. Missed opportunities for diagnosis (MODs) were defined as those episodes of medical care where IBD diagnostic workup was not started despite the presence of one or more signs/symptoms suggestive of IBD being reported during the interview.

Medical care episodes were defined as those situations of doctor–patient interaction either in primary care, emergency care departments (ERs), or hospital care (4). The exact dates of the onset of symptoms and the initial consultation and the type of physician were collected, as well as the number of visits to the same physician before being referred to the PG, number of physicians consulted until reaching an IBD diagnosis, number of visits to the ER and hospital admissions generated before the diagnosis, date of referral to the PG, date of the first visit to the PG, date of the endoscopic procedures, and the date of IBD diagnosis if they were not the same.

The IBD phenotype was recorded according to the Paris classification (13). Disease activity at diagnosis was calculated using the weighted Pediatric Crohn's Disease Activity Index (wPCDAI) (14) for CD. The Pediatric Ulcerative Colitis Activity Index (PUCAI) (15) was used to calculate UC activity. We considered C-reactive protein (CRP), erythrocyte sedimentation rate, albumin, complete blood count, and fecal calprotectin, although orosomucoid levels do not have the sensitivity of CRP or calprotectin was used as a laboratory marker in some centers and was also collected (16). Anthropometric (17), epidemiological data (rural or urban environment; a population of over 10,000 inhabitants was considered urban), and smoking status (active or passive) were also collected. Both passive and active exposures to tobacco smoke in childhood affect the development of IBD, although the effects on it are more evident with active smoking than with passive smoking (18).

To consider the time of onset of symptoms, the following were considered signs or symptoms suggestive of IBD (4):

- Signs: recurrent mouth ulcers, recurrent perianal disease (abscesses, fistulae, fissures, or skin tags), prolonged fever, extraintestinal manifestations (episcleritis, scleritis, uveitis, erythema nodosum, gangrenous pyoderma, psoriasis, arthritis, or digital clubbing), delayed linear growth (at least 1 standard deviation below the target height), or delayed puberty.
- Symptoms: asthenia or anorexia, nocturnal bowel movements, urgency, diarrhea lasting ≥2–4 weeks (Bristol stool chart type 5–7) or ≥2 episodes in the past 6 months with no other apparent cause, bloody diarrhea lasting >1 week, recurrent abdominal pain >14 days or ≥2 episodes of abdominal pain in the past 5 months (not meeting Rome IV criteria for a functional disorder), unintended weight loss >1 kg, or anal bleeding in the absence of constipation (based on Rome IV criteria).

### Statistical Analysis

Variables with normal distribution were expressed as mean ± standard deviation, and those without normal distribution were expressed as median and interquartile range (IQR). Kolmogorov–Smirnov test was used to evaluate the normality of the distribution. Student's *t*-test and Wilcoxon signed-rank test were used for paired samples, and the Chi-square test was used to compare proportions. To compare variables, the Kruskal–Wallis test was applied. If the hypothesis of equality was rejected, groups were compared using the Mann–Whitney's *U*-test with Bonferroni correction. Times to diagnosis were compared with Kaplan–Meier curves and the log-rank test. Predictive models were constructed using univariate and multivariate logistic regression tests. The variables that present statistically significant differences or a trend (*p* < 0.15) in the univariate analyses, together with the variables that, due to theoretical or empirical knowledge, are considered to be related to the dependent variable, will be used for the construction of the model. The magnitude of the association between the predictive variables of the model and the dependent variable will be measured using the odds ratio and their corresponding 95% confidence interval. Statistical analysis was performed using SPSS (IBM SPSS Statistics for Mac, version 22.0) and GraphPad Prism Version 7.04. A two-sided *p*-value < 0.05 was considered statistically significant.

### Ethical Issues

The study and protocols for recruitment were approved by the Ethics Committee of the Principado de Asturias, reference number 106/2012.

## Results

Study sample comprised a total of 149 patients from 24 hospitals, 97 (65.1%) with CD, 48 (32.2%) UC, and 4 (2.7%) IBD-U. One hundred and thirty-one patients (87.9%) lived in an urban area, 27 (18.1%) had a family history of IBD, and 19 (12.8%) were passive smokers. Only one patient previously had an appendectomy. Clinical characteristics of CD and UC patients are summarized in Table 1.

**Table 1:** Clinical characteristics of study subjects (*n* = 145).

**Variable**	**n (%)**		
Male	89 (61.3%)		
Age at diagnosis (years)	11.2 ± 2.9		
**Anthropometry at diagnosis**^**2**^**, median IQR**	**CD**	**UC**	**p**
Weight (kg) Weight z score Height (cm) Height z score	38.4 (30.2–45.8) −0.5 (−1.03–0.01) 150 (137–158) −0.14 (−0.84–0.6)	42.8 (31.8–53.0) −0.12 (−0.91–0.71) 154 (139.2–161.7) 0.21 (−0.37–1.29)	0.053 0.096 0.229 0.039
**Paris classification**			
Ulcerative colitis (n = 48)			
E1 E2 E3 E4 S0 S1^1^	7 (15.6%) 6 (13.3%) 9 (20%) 23 (51.1%) 39 (81.2%) 9 (18.8%)		
Crohn's disease (n = 97)			
L3 L3L4a L1 L2 L1L4a L4b L2L4a L4a L3L4ab L3L4b L4ab B1 B2 B3 Perianal disease (p) Growth retardation (G1)	37 (38.1%) 20 (20.6%) 17 (17.5%) 7 (7.2%) 4 (4.1%) 3 (3.1%) 3 (3.1%) 2 (2.1%) 2 (2.1%) 1 (1.0%) 1 (1.0%) 88 (90.7%) 5 (5.2%) 4 (4.1%) 18 (18.5%) 17 (17.5%)		
**Disease activity at diagnosis**			
Disease activity UC (PUCAI)^3^, median (IQR) Mild Moderate Severe	40 (25–50) 18 (37.5%) 23 (47.9%) 7 (14.6%)		
Disease activity CD (wPCDAI)^4^, median (IQR) Remission Mild Moderate Severe	47.5 (39–60) 3 (3.1%) 33 (34%) 30 (30.9%) 31 (32%)		
**EIM**	23 (15, 4%)		
**Laboratory parameters at diagnosis**	**CD**	**UC**	**P**
Faecal calprotectin (μg/g)	500 (291–880)	500 (304–1,450)	0.405
CRP (mg/dl)	2.31 (0.8–7.0)	0.5 (0.2–1.7)	0.0001
ESR (mm/h)	36 (22–62)	24 (8–41)	0.004
Hb (g/dl)	11.5 (10.6–12.2)	11.8 (9.6–13.1)	0.818
Htc (%)	35.4 (33.0–38.0)	34.5 (30.1–40.7)	0.877
Platelets (×10^9^/L)	477 (376–573)	379 (298–481)	0.001
Orosomucoid (mg/L)	234 (145–304)	105 (78–124)	0.13

1 PUCAI ≥65 points.

2 Reference values (16). Paris classification was adapted from Levine et al. (13). EIM, extraintestinal manifestations.

3 PUCAI, Paediatric Ulcerative Colitis Index; Remission <10; Mild 10–34; Moderate 35–64; Severe ≥65 points. From reference (15).

4 *wPCDAI, weighted Pediatric Crohn's Disease Activity Index; Remission <12.5; Mild 12.5–40; Moderate >40; Severe >57.5 points. From reference (14); CD, Crohn's disease; UC, ulcerative colitis; CRP, C-reactive protein; Hb, hemoglobin; Htc, hematocrit; ESR, Erythrocyte sedimentation rate; EIM, extraintestinal manifestations; IQR, Interquartile range. The four patients with inflammatory bowel disease unclassified were excluded from the analysis*.

**Figure 1:**
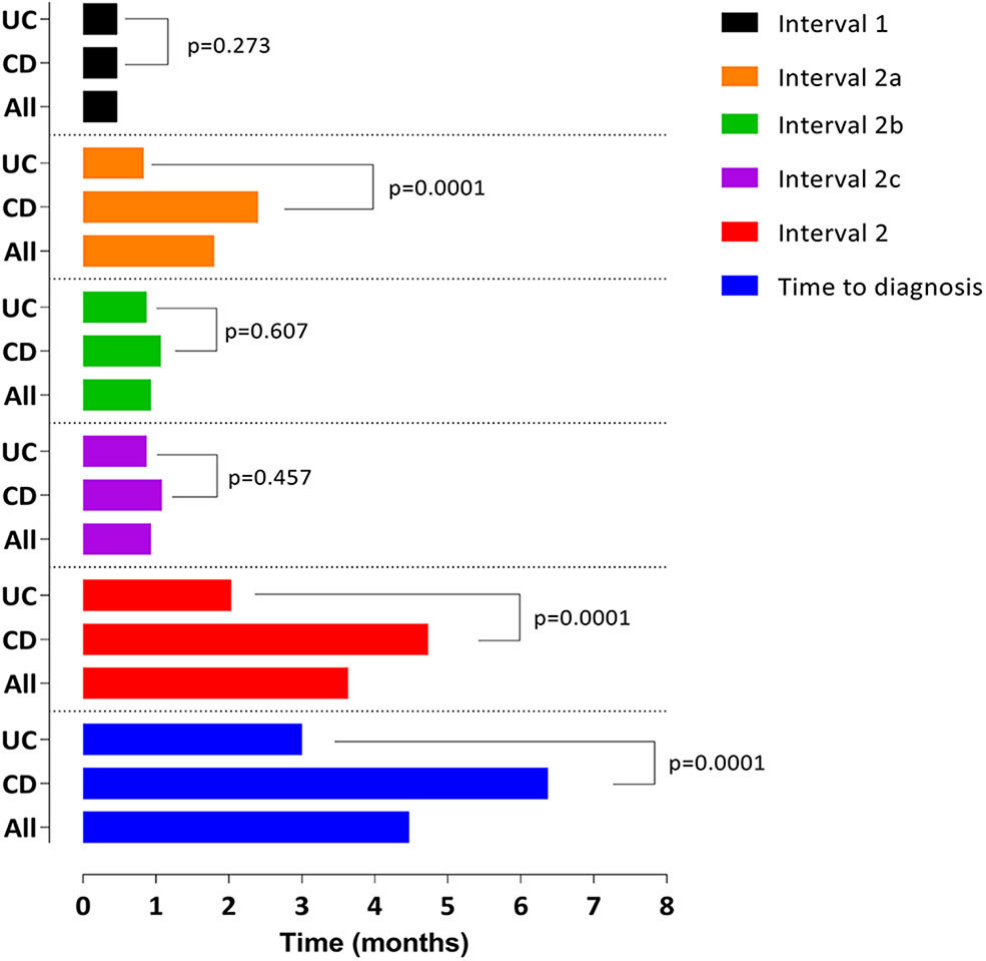
Graphical representation of the median duration (months) of time to diagnosis and the corresponding subintervals. **Interval 1 (days):** All: 13.8 (6.9–32.4), CD: 13.8 (6.9–57.9), UC 13.8 (7.2–30.6), *p* = 0.273. **Interval 2a (months):** All: 1.8 (0.66–5.06), CD: 2.4 (1.03–7.17), UC: 0.83 (0.30–2.50), *p* = 0.0001. **Interval 2b (days):** All: 7 (1–31), CD: 7.5 (1–31), UC: 7 (1–29), *p* = 0.607. **Interval 2c (days):** All: 14 (6–34), CD: 14 (6–49), UC: 14 (5–29), *p* = 0.457. **Interval 2 (months):** All: 3.6 (1.8–8.7), CD: 4.7 (2.4–9.8), UC: 2.0 (1.0–4.9), *p* = 0.0001. **Time to diagnosis (months):** All: 4.4 (2.6–10.4), CD: 6.3 (3.3–12.3), UC: 3 (1.6–5.6), *p* = 0.0001.

Time from symptom onset to first physician consultation (interval 1) was 13.8 days (6.9–32.4), without differences between CD and UC (13.8 days [IQR 6.9–57.9] vs. 13.8 [IQR 7.2–30.6], *p* = 0.273) (Figure 1). No significant association with the studied variables was observed (Supplementary Table 1).

**Figure 2:**
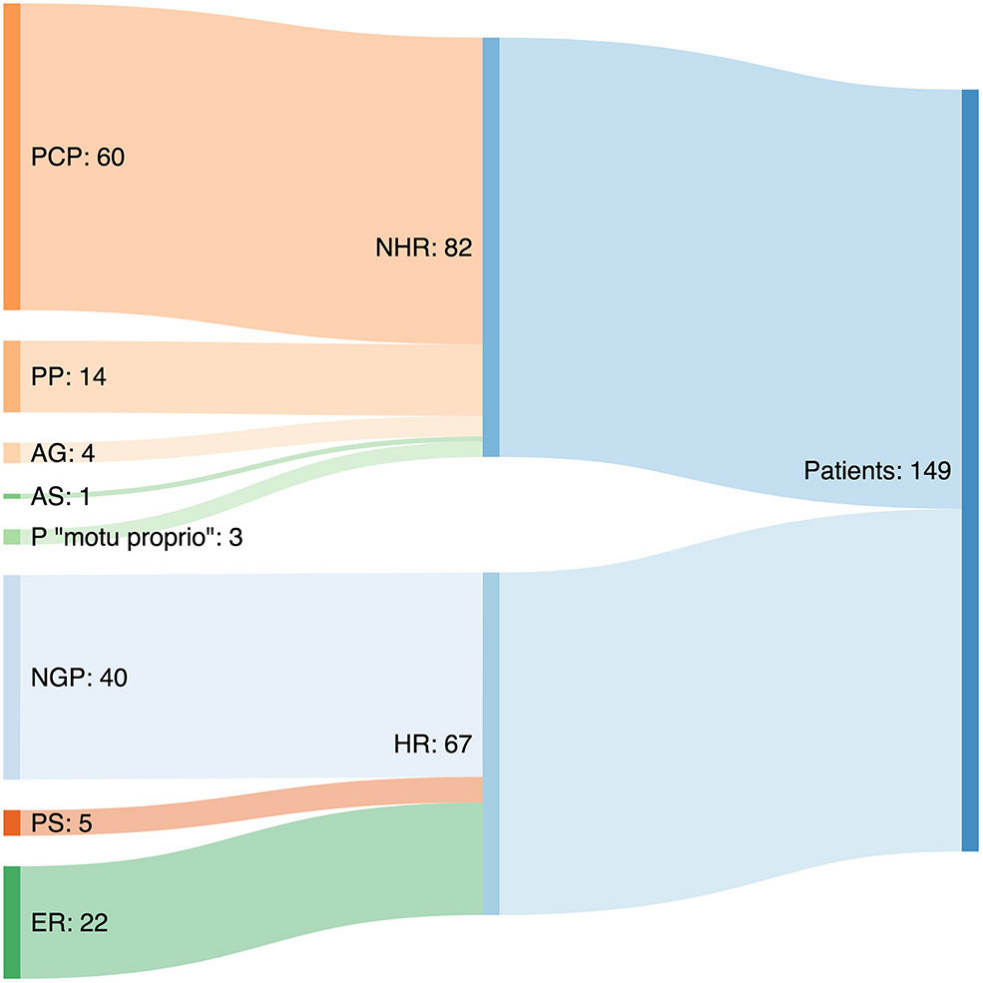
Sankey diagram showing the routes of referral of patients to the pediatric gastroenterologist. PCP, primary care pediatrician; PP, private pediatrician; ER, referrals from emergencies; AS, adult specialist; AG, adult gastroenterologist; P, parents “motu proprio”; NGP, non-gastroenterologist pediatrician; PS, pediatric surgeon; HR, hospitalized referrals; NHR, non-hospitalized referrals http://sankeymatic.com/build/.

Regarding interval 2a (time from the first physician's visit until referral to a PG), and in considering the first physician visited, 74.5% of the patients went to their PCP, 12.8% to the ER, 8.7% to a private pediatrician, and the remaining 4% to other types of physicians (adult PG, general practitioner, surgeon, etc.). Of the 149 patients, 110 (73.8%) visited more than one physician (43.5% to two, 20.4% to three, and 29.9% to four or more) before being assessed by the PG. Considering those patients who were seen by a second physician, more than half (59.8%) went to ER, 10.3% went to a PCP, the same percentage was seen by a pediatrician in an outpatient hospital clinic (10,3%), an additional 8.4% went to a pediatrician working in private practice, and the remaining 11.2% went to another type of physician. The way the patients reached the PG clinic is depicted in Figure 2. Patients' diagnoses previous to IBD are shown in Table 2.

**Table 2:** Diagnoses before the IBD diagnosis.

**Diagnoses**	**Episodes**
Acute gastroenteritis Chronic diarrhea Perianal abscess/Anal fissure Recurrent abdominal pain Gastritis Hemorrhoids Lactose intolerance Irritable bowel syndrome Food allergy/intolerance Celiac Disease Anemia Anorexia nervosa Rheumatism Non-gastrointestinal infection Short stature Rectal bleeding Rectal prolapse	62 23 16 12 7 6 5 5 4 4 4 3 2 2 1 1 1

The number of visits to the same physician before referral was one in 20.4%, two to three (47.7%), and four to six (16.3%). An additional 16.4% attended more than six times. There were no significant differences in relation to presenting signs and symptoms and a greater number of visits to the PCP or more doctors, except for weight loss (Supplementary Table 2).

Referral to PG was performed by PCP in 40.3% of cases, by non-PG pediatricians (NGP) (26.8%), by ER pediatricians (14.8%), by pediatricians in private practice (9.4%), and by other doctors in the remaining 8.7%. Regarding hospital admissions before diagnosis, 22 (14.7%) patients required one admission and five (3.3%) patients two or more (Supplementary Table 3).

**Figure 3:**
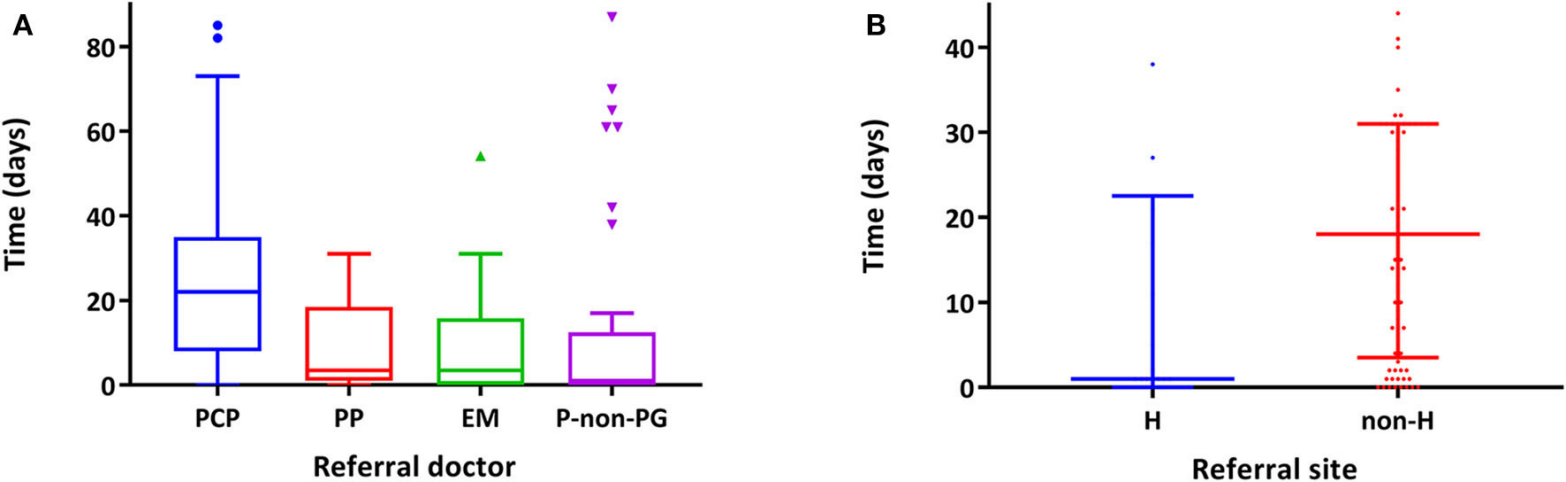
Interval 2b, time (days) from referral to the PG until the PG office visit. **(A)** Differences according to the professional who makes the referral (*p* = 0.0001). Only those professionals who have made five or more referrals are included. PCP, primary care pediatrician; PP, private pediatrician; ER, emergencies; NGP, non-gastroenterologist pediatricians. **(B)** Differences according to whether or not the patient was hospitalized during the referral (*p* = 0.0001). H, hospitalized; Non-H, not hospitalized.

In relation to interval 2b, there were significant differences in relation to the doctor who referred the patient (Figure 3) being significantly higher in those referred by a PCP followed by a pediatrician in private practice, pediatricians from ER, and, lastly, by NGP working at a hospital (22 days [RIQ 8–35] vs. 3.5 days [RIQ 1–18.5] vs. 3.5 days [RIQ 0–15.75] vs. 1 day [RIQ 0–6]; *p* = 0.0001). There were also differences if the referral was made while the patient was hospitalized or on an outpatient basis (0 days [RIQ 0–1] vs. 21 days [RIQ 7–34], *p* = 0.001), although admitted patients had higher scores in the activity indices (wPCDAI: 62.5 [RIQ 47–78] vs. 45 [RIQ 36–56], *p* = 0.003; PUCAI: 45 [RIQ 42–70] vs. 30 [RIQ 20–45], *p* = 0.003). In this sense, referral by pediatricians from hospital care (NGP and ER) compared with referral from primary care or private practice pediatricians allowed earlier IBD diagnosis (odds ratio 3.2 [95% confidence interval 1.1–8.9], *p* = 0.025).

Interval 2c duration did not change depending on the final diagnosis (Supplementary Table 4). Endoscopy for diagnosis was carried out in 70.3% of cases by a PG, in 15.2% by the pediatric surgeon, and by an adult gastroenterologist in the remaining 14.5%. There were no differences in the duration of interval 3 regarding the professional who performed the endoscopic procedure (14 days [IQR 6–43] vs. 14.5 [IQR 4–36] vs. 12 days [IQR 4–28], *p* = 0.651). Predictors of interval 3 duration below the 75th percentile are shown in Table 3.

**Table 3:** Variables predicting an interval 2c lower than P75.

**Variable**	**Univariate OR (CI 95%)**	***p***	**Multivariate OR (CI 95%)**	***p***
EIM	8.9 (1.1–68)	0.036		
CRP > 2 mg/dl	2.35 (1.02–5.4)	0.044		
Severe disease	5.6 (1.6–19.6)	0.007	5.8 (2.1–15.9)	0.003
Faecal calprotectin > 500 mcg/g	4.6 (1.8–11.7)	0.049	12.2 (2.6–56.8)	0.003

*Hosmer and Lemeshow test: p = 0.875; Cox–Snell R^2^: 0.197. Nagelkerke R^2^: 0.294; Sensitivity: 51 (35–66); Specificity 88 (82–95); PPV: 70 (53–86); NPV: 78 (69–86). The model displayed here is significant, explains between 0.197 and 0.294 of the dependent variable, and correctly classifies 88.9% of cases. IBD, inflammatory bowel disease; EIM, extraintestinal manifestations; CRP, C-reactive protein*.

There were 661 MODs in 145 patients, corresponding to a median of 3 MODs (IQR 1–7) per patient. The number of MODs was significantly higher in CD than that in UC patients: 4 MODs (RIQ 2–7) vs. 2 MODs [RIQ (1–5), *p* = 0.003] (Table 4).

**Table 4:** Variables predicting an interval 2 lower than P75.

**Variable**	**Univariate OR (CI 95%)**	***p***	**Multivariate OR (CI 95%)**	***p***
Severe disease	0.47 (0.17–1.21)	0.116		
Perianal disease (EC)	0.214 (0.044–1.038)	0.056		
Fecal calprotectin (UC)	14.2 (1.59–127)	0.017		
Referral during admission	2.3 (1.03–7.09)	0.042	2.78 (1.032–7.501)	0.043
MODs	0.87 (0.79–0.84)	0.002	0.86 (0.79–0.94)	0.002

*Hosmer and Lemeshow test: p = 0.421; Cox–Snell R^2^: 0.104. Nagelkerke R^2^: 0.155; Sensitivity: 13 (2.5–25); Specificity 96 (92–98); PPV: 55 (23–88); NPV: 77 (69–84). This table only shows the results of the univariate analysis of the variables that were finally included in the multivariate analysis. The model displayed here is significant, explains between 0.104 and 0.155 of the dependent variable, and correctly classifies 75.7% of cases. IBD, inflammatory bowel disease; MODs, missed opportunities for diagnosis*.

Interval 2 was significantly higher in CD patients: 4.7 months (IQR 2.4–9.8) vs. 2.0 (1.0–4.9), *p* = 0.0001. Referral during admission and lower number of MODs contributed significantly to the reduction of this interval (Table 4, Supplementary Table 5).

**Figure 4:**
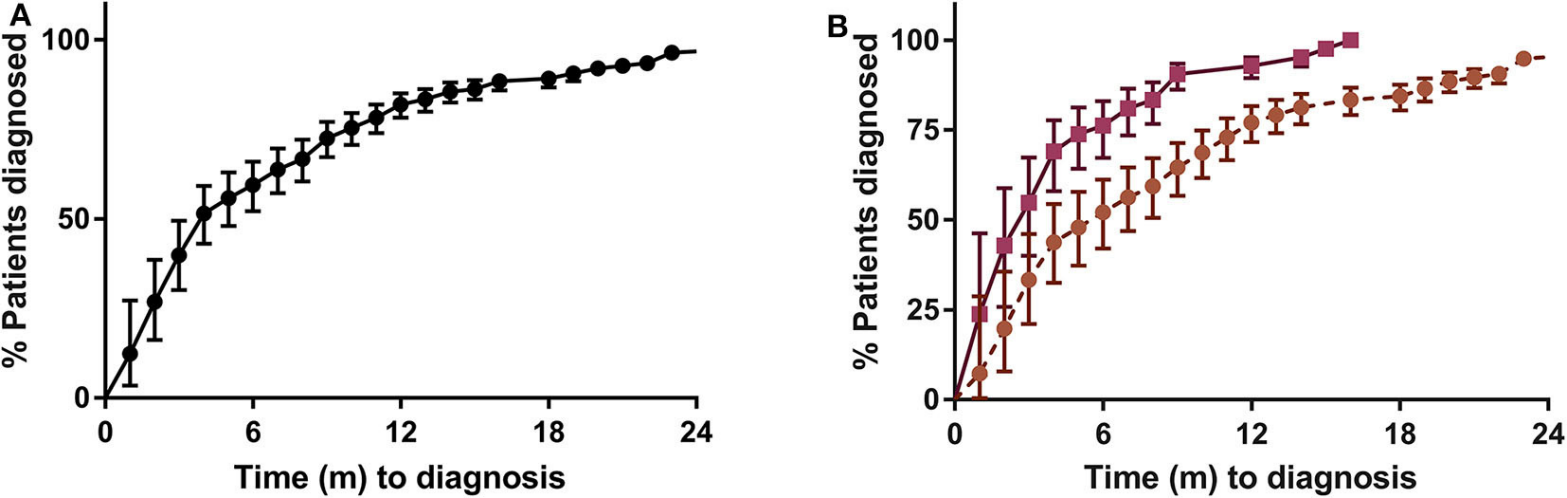
Kaplan–Meier survival curve. **(A)** All the samples (*n* = 145). **(B)** CD (circular marks, five data points are outside the axis limits) vs. UC (square marks), *p* = 0.0001.

Overall TD was 4.4 months (IQR 2.6–10.4) (Figure 1) being significantly higher in CD than in UC patients (6.3 [RIQ 3.3–12.3] vs. 3 [RIQ 1.6–5.6] months, *p* = 0.0001). Interval 2a was the main contributor to TD (2.4 months [IQR 1.03–7.17] in CD vs. 0.83 months [IQR 0.30–2.50] in UC, *p* = 0.0001). A graphical representation of the percentage of patients diagnosed in the 2 years after the onset of symptoms is shown in Figure 4.

## Discussion

Our study shows that TD in pediatric IBD in Spain is longer for CD than for UC, as it has been observed in pediatric and adult series from other countries (3, 5, 9–11, 17–28). It has also been demonstrated that the majority of this delay is generated in the timespan from the first consultation with a physician, who is usually the PCP, to the referral to PG. Our study, unlike those previously published, identifies six different intervals allowing the identification of the interval where the highest DD is generated.

Interval 1, in which responsibility lies with the patient and/or family, is usually unknown and is divided into two subintervals: one from the moment that the disease is established until the patient is aware of the symptoms and shares them with their parents and, subsequently, from that moment until they seek medical advice. Factors contributing to delay in this interval are lack of parent/patient recognition of signs/symptoms (e.g., growth failure, fatigue, mild abdominal pain, etc.), shame due to digestive symptoms (diarrhea, perianal discomfort, etc.), or anxiety caused by fear of receiving bad news. Other factors could be the patient's social environment, accessibility to health care facilities, self-medication, the belief that it may be a transitory condition, personal beliefs, and the use of alternative medicines to mitigate/alleviate the symptoms.

In our series, there were no differences between CD and UC, with a median interval 1 for both entities of 13 days. There were no differences either regarding the positive family history of IBD. Factors contributing to the delay in the consultation could not be identified through our analysis. Our data can be compared with a Swiss cohort (3, 5), where patients took a little longer to visit a physician than in our series (median 1 month) and with a study conducted in Saudi Arabia (22) where, curiously, interval 1 was higher than interval 2, the one attributable to the health system.

Interval 2a, from the first consultation with the doctor to the referral to the PG, is of utmost importance and is the one on which actions should be focused. This interval is generally quantified in months and is of special relevance, as it has been identified as the main contributor to TD. The three determining factors of this interval are the index of suspicion of IBD, communication routes with the reference unit to which the patient should be referred, and the physician's previous experiences with the disease. In the univariate analysis, perianal involvement was associated with diagnostic delay, although it was not statistically significant. This may be due to various reasons such as embarrassment, lack of relationship with IBD, or that anal inspection is not a common practice in primary care unless the patient reports specific symptoms.

Repeated visits to the same doctor and to different doctors for the same symptoms deserve special mention. Perhaps, the most successful approach in these situations is to know the periods of usual evolution of the different diagnosed entities and transmit this to families and, in case the symptoms persist beyond the established period, start the differential diagnosis (4). It is worth noting the high number of MODs in our series, an aspect that has not been assessed or quantified in other published series (4). MODs are instances in which *post-hoc* judgment indicates that alternative decisions or actions could have led to a more timely diagnosis. It should be emphasized that not all missed opportunities or delays necessarily result in harm or poor patient outcomes and not all instances of delayed diagnosis are associated with missed opportunities. MODs may occur with any disease, but in the literature, most papers where MODs are analyzed are about cancer (29). This is an aspect that has not been assessed or quantified in other published series of pediatric IBD.

In relation to interval 2b, a purely administrative interval, we have observed that those patients referred by professionals other than PCPs and patients referred to the pediatric gastroenterology clinic during admission were assessed earlier by the PG. This aspect is of utmost importance, as PCPs, in the majority of regions in Spain, do not have a way to accelerate the visit with the PG after the referral, which in addition, in many regions, is subject to a maximum time of response of 60 days. Other routes of contact with the referral unit should be promoted to speed up the assessment by PG.

Regarding interval 2c (time from the PG visit until IBD diagnosis), there were no significant differences related to the final diagnosis (CD vs. UC). No differences were observed regarding the type of physician responsible for performing the endoscopic procedure. Severity of the flare (wPCDAI/PUCAI) and higher fecal calprotectin values played a relevant role in early diagnosis, maybe through increasing the index of suspicion by the PG. Hospital access to perform endoscopy did not play any relevant role in the duration of this interval, as it is not an obstacle in our country. Interval 2c values in our series are comparable with a series from Canada (10), where the time between the PG receiving the patient and performing the endoscopy is, as in our study, 2 weeks.

Interval 2 length (from the patient's first visit to the physician until diagnosis) is mainly dependent on the health system. It was clearly influenced by two variables: the MODs and referral from hospital admission. We found no study about pediatric IBD analyzing MODs, so we cannot compare our results.

It is not uncommon in our country that, in the absence of a diagnosis from PCPs in a situation of persisting symptoms, a patient goes to the emergency services trying to speed up the diagnostic process through hospital admission. It should be highlighted that to reduce the time to reach an adequate IBD diagnosis, work must also be done in the hospital setting (emergencies, hospitalization wards, and outpatient clinics).

The most significant contribution of our work, besides knowing the situation of the TD in Spain, is to identify the link in the chain where the greatest delay is located, to be able to work specifically on improving the weaknesses of that link. No previous published studies divide the TD into six intervals. This division allowed a more careful assessment of the weight of each in the diagnostic delay. We have demonstrated the large number of medical visits that an IBD patient undergoes before being diagnosed, a fact that no other study has analyzed before. Although the prospective design makes the data more reliable, our study has several limitations. One of the limitations of the present study could be the number of patients included. However, we consider that the number and geographic distribution of the hospitals allow us to make valid conclusions, and the results of the present study can be extrapolated. Although it was performed prospectively as soon as patients are diagnosed, the fact that the TD is long implies that the reliability of the data depending on parents' recollection might not be accurate. Data provided by the family have not been contrasted with patients' medical records, so when using only those reported by the family, we could have a collection bias, as relatives might magnify the TD, thus blaming the physician. Finally, and now that we have identified that the interval where the highest TD is generated is in the hands of the PCP, it is worth evaluating different strategies to improve this. The first, and the one which we have been implementing since we analyzed these results, is to provide information to PCP about IBD. Due to its infrequent nature at the pediatric age, many pediatricians do not take this entity into account in their differential diagnosis. On the other hand, the establishment of protocols for blood tests (CRP, albumin, iron profile, etc.) and fecal markers (calprotectin being the most available test in general practice) increases the suspicion of a possible IBD. And finally, fast and specific referral channels for this type of patient (4) are needed.

Our study confirms that TD in pediatric IBD in Spain is greater for CD than for UC, as was observed in series from other countries, and identifies that the interval where the highest delay is generated (interval 2a) starts when the patient comes to his first doctor visit, who is usually a PCP, until he is referred to the PG.

## Data Availability Statement

The raw data supporting the conclusions of this article will be made available by the authors, without undue reservation.

## Ethics Statement

The studies involving human participants were reviewed and approved by Ethics Committee of the Principado de Asturias, reference number 106/2012. Written informed consent to participate in this study was provided by the participants' legal guardian/next of kin.

## Author Contributions

SJ designed the data collection instruments, coordinated and supervised data collection, conducted the analyses, drafted the initial manuscript, and reviewed and edited the manuscript. VMN-L, JM, GP, JD, and RM-M conducted the analyses, drafted the initial manuscript, and reviewed and edited the manuscript. AR, OS, LP, HA, FE, JB, JG, LO, ED, VC, PB, GB, JB, MJ, CO, ZG, GG, PO, HL, RG, EL, and DP reviewed and edited the manuscript and all authors approved the final manuscript as submitted and agree to be accountable for all aspects of the work. All authors have read and agreed to the published version of the manuscript.

## Conference Presentation

This paper was presented as a poster at the 4th International Symposium on Pediatric Inflammatory Bowel Disease, 13th−16th September 2017, Barcelona, Spain.

## Conflict of Interest

The authors declare that the research was conducted in the absence of any commercial or financial relationships that could be construed as a potential conflict of interest.
