# Supplementary material for: Spanish Pediatric Inflammatory Bowel Disease Diagnostic Delay Registry: SPIDER Study From Sociedad Española de Gastroenterología, Hepatología y Nutrición Pediátrica
Source: Front Pediatr. 2020 Oct 15;8:584278. doi: 10.3389/fped.2020.584278 (PMC7593447; doi:10.3389/fped.2020.584278)
Supplement: Supplementary file 1 [file Table_1.DOCX]

| **Table S1. Interval 1. Hypothesis contrast** | | | | | | | | | |
| --- | --- | --- | --- | --- | --- | --- | --- | --- | --- |
|  | **Total** | | | **CD** | | | **UC** | | |
| **Variable** | **PI** | **nPI** | **p** | **PI** | **nPI** | **p** | **PI** | **nPI** | **p** |
| Gender (Female), % | 42.9 | 40.0 | 0.457 | 30.4 | 33.8 | 0.448 | 66.7 | 52.8 | 0.310 |
| Family history, % | 23.5 | 16.4 | 0.239 | 30.4 | 16.2 | 0.117 | 9.1 | 16.7 | 0.473 |
| Rural zone, % | 14.3 | 11.8 | 0.448 | 13.0 | 10.8 | 0.511 | 16.7 | 13.9 | 0.569 |
| Diarrhea, % | 37.1 | 37.3 | 0.577 | 39.1 | 41.9 | 0.506 | 33.3 | 27.8 | 0.489 |
| Abdominal pain, % | 20.0 | 35.5 | 0.064 | 30.4 | 40.5 | 0.268 | 0.0 | 25.0 | 0.056 |
| Rectal bleeding, % | 22.9 | 17.3 | 0.305 | 4.3 | 5.4 | 0.661 | 41.7 | 58.3 | 0.251 |
| Perianal disease, % | - | - | - | 4.3 | 8.1 | 0.472 | - | - | - |
| EIM, % | 17.1 | 15.9 | 0.523 | 17.4 | 18.1 | 0.607 | 16.7 | 11.4 | 0.486 |
| Severe disease (wPCDAI/PUCAI),% | 33.3 | 27.9 | 0.347 | 31.8 | 36.3 | 0.443 | 34.6 | 9.1 | 0.054 |
| Z score weight, median IRQ | -0.53 (-1.1- -0.06) | -0.33 (-0.93-0.62) | 0.303 | -0.58 (-1.1- -0.12) | -0.47 (-0.99-0.16) | 0.495 | -0.49 (-1.2-0.6) | -0.05 (-0.91-0.83) | 0.446 |
| Z score height, median IQR | -0.17 (-1.38-0.95) | 0.03 (-0.64-0.82) | 0.197 | -0.34 (-1.4-0.95) | -0.13 (-0.79-0.59) | 0.481 | -0.06 (-1.4-1.03) | 0.46 (-0.36-1.3) | 0.243 |
| Prolonged Interval was defined as an overall time greater than P75. Interval 1: Total: 32 days; CD: 58 days; UC: 30 days. PI: prolonged interval. nPI: no prolonged interval. CD: Crohn’s disease. UC: Ulcerative colitis. CRP: C-reactive protein; Hb: haemoglobin; Hct: haematocrit; ESR: Erythrocyte sedimentation rate; EIM: extraintestinal manifestations; IQR: Interquartile range. The 4 patients with IBDU were excluded from the analysis | | | | | | | | | |

| Table S2. Signs and symptoms related with a higher number of visits to the same doctor and with different doctors before being referred to PG. | | | | | | |
| --- | --- | --- | --- | --- | --- | --- |
| Sign/symptom (n/%) | Number of visits to the PCP≤ 4 (n=112) | Number of visits to the PCP> 4 (n=33) | p value | Number of doctors visited before PG ≤ 3 (n=131) | Number of doctors visited before PG > 3 (n=14) | p value |
| Abdominal pain | 36 / 32.1% | 4 / 28.6% | 0.511 | 42 / 32.1% | 4 / 28.6% | 0.526 |
| Diarrhea | 38 / 33.9% | 5 / 35.7% | 0.095 | 49 / 37.4% | 5 / 35.7% | 0.574 |
| Rectal bleeding | 23 / 20.5% | 1 / 7.1% | 0.204 | 26 / 19.8% | 1 / 7.1% | 0.220 |
| Weight loss | 5 / 4.5% | 3 / 21.4% | 0.269 | 2 / 1.5% | 3 / 21.4% | 0.006 |
| Growth failure | 0 / 0% | 0 / 0% | 0.228 | 1 / 0.8% | 0 / 0% | 0.903 |
| EIM | 2 / 1.8% | 0 / 0% | 0.595 | 2 / 1.5% | 0 / 0% | 0.816 |
| Perianal disease | 6 / 5.4% | 1 / 7.1% | 0.499 | 6 / 4.6% | 1 / 7.1% | 0.517 |
| Anaemia | 2 / 1.8% | 0 / 0% | 0.595 | 2 / 1.5% | 0 / 0% | 0.816 |
| Fever | 0 / 0% | 0 / 0% | 0.228 | 1 / 0.8% | 0 / 0% | 0.903 |
| The median number of visits to the PCP before being referred to the GP was 3 (IQR, 2-4) and the median number of doctors visited before being assessed by the GP was 2 (IQR 1-3). EIM: extraintestinal manifestations. PCP: Primary care paediatrician. PG: Pediatric Gastroenterologist | | | | | | |

| **Table S3. Interval 2a. Hypothesis contrast** | | | | | | | | | | |
| --- | --- | --- | --- | --- | --- | --- | --- | --- | --- | --- |
|  | **Total** | | | **CD** | | | | **UC** | | |
| **Variable** | **PI** | **nPI** | **p** | **PI** | **nPI** | **p** | **PI** | | **nPI** | **p** |
| Gender (Female), % | 34.3 | 42.6 | 0.252 | 29.2 | 33.3 | 0.456 | 45.5 | | 61.1 | 0.283 |
| Family history, % | 11.4 | 20.6 | 0.169 | 12.5 | 22.2 | 0.235 | 9.1 | | 17.1 | 0.459 |
| Rural zone, % | 5.7 | 14.8 | 0.129 | 0.0 | 15.3 | 0.034 | 18.2 | | 13.9 | 0.527 |
| Diarrhea, % | 31.4 | 38.9 | 0.279 | 33.3 | 44.4 | 0.238 | 27.3 | | 27.8 | 0.647 |
| Abdominal pain, % | 25.7 | 33.3 | 0.266 | 33.3 | 38.9 | 0.408 | 9.1 | | 22.2 | 0.313 |
| Rectal bleeding, % | 8.6 | 3.7 | 0.229 | 4.2 | 5.6 | 0.633 | 45.5 | | 47.2 | 0.597 |
| Perianal disease, % | 17.1 | 19.4 | 0.489 | 12.5 | 5.6 | 0.238 | - | | - | - |
| EIM, % | 15.2 | 16.8 | 0.530 | 13.6 | 19.4 | 0.395 | 18.2 | | 11.4 | 0.445 |
| Severe disease (wPCDAI/PUCAI),% | 24.2 | 31.4 | 0.292 | 26.1 | 39.1 | 0.191 | 20.0 | | 15.2 | 0.524 |
| Z score weight, median IRQ | -0.34 (-0.9-0.4) | -0.41 (-0.98-0.54) | 0.767 | -0.49 (-0.99- -1.014) | -0.53 (-1.04-0.12) | 0.692 | 0.16 (-0.71-0.87) | | -0.05 (-0.92-0.71) | 0.911 |
| Z score height, median IQR | -0.19 (-0.89-0.59) | -0.03 (-0.81-0.85) | 0.666 | -0.26 (-0.81-0.59) | -0.13 (-0.86-070) | 0.705 | 0.49 (-1.15-1.32) | | 0.15 (-0.37-1.29) | 0.970 |
| PCP first Physician | 82.9 | 74.1 | 0.205 | 95.8 | 77.8 | 0.036 | 54.5 | | 66.7 | 0.349 |
| Prolonged Interval was defined as an overall time greater than P75. Interval 2: Total: 5 months; CD: 7 months; UC: 2,5 months. PI: prolonged interval. nPI: no prolonged interval.. CD: Crohn’s disease. UC: Ulcerative colitis. CRP: C-reactive protein; Hb: haemoglobin; Hct: haematocrit; ESR: Erythrocyte sedimentation rate; EIM: extraintestinal manifestations; IQR: Interquartile range. The 4 patients with IBDU were excluded from the analysis | | | | | | | | | | |

| **Table S4. Interval 2c. Hypothesis contrast** | | | | | | | | | |
| --- | --- | --- | --- | --- | --- | --- | --- | --- | --- |
|  | **All** | | | **CD** | | | **UC** | | |
| **Variable** | **DD** | **nDD** | **p** | **DD** | **nDD** | **p** | **DD** | **nDD** | **p** |
| Gender (Female), % | 33.3 | 43.5 | 0.190 | 33.3 | 32.9 | 0.577 | 33.3 | 65.7 | 0.053 |
| Family history, % | 25 | 15.9 | 0.164 | 16.7 | 20.5 | 0.465 | 41.7 | 5.9 | 0.009 |
| Rural zone, % | 11.1 | 13.0 | 0.515 | 12.5 | 11.0 | 0.545 | 8.3 | 17.1 | 0.417 |
| Diarrhea, % | 36.1 | 37.0 | 0.543 | 33.3 | 43.8 | 0.254 | 41.7 | 22.9 | 0.187 |
| Abdominal pain, % | 36.1 | 30.6 | 0.336 | 37.5 | 38.4 | 0.570 | 33.3 | 14.3 | 0.153 |
| Rectal bleeding, % | 16.7 | 19.4 | 0.461 | 12.5 | 2.7 | 0.095 | 25.0 | 54.3 | 0.077 |
| Perianal disease, % | - | - | - | 16.7 | 4.1 | 0.061 | - | - | - |
| EIM, % | 2.9 | 20.8 | 0.008 | 0 | 23.6 | 0.005 | 8.3 | 14.7 | 0.5 |
| Severe disease (wPCDAI/PUCAI),% | 9.1 | 35.9 | 0.002 | 8.7 | 44.3 | 0.001 | 10.0 | 18.2 | 0.476 |
| Z score weight, median IRQ | -0.35 (-0.67-0.62) | -0.47 (-0.99-0.33) | 0.504 | -0.54 (-1.03- -0.3) | -0.5 (-1.0-0.06) | 0.738 | 0.49 (-0.46-1.1) | -0.31 (-0.93-0.68) | 0.130 |
| Z score height, median IQR | 0.08 (-0.43-0.8) | -0.13 (-0.87-0.85) | 0.475 | -0.08 (-0.63-0.57) | -0.2 (-0.8-0.7) | 0.682 | 0.66 (-0.32-1.2) | 0.15 (-0.83-1.31) | 0.558 |
| Faecal calprotectin (μg/g) | 313 (216-500) | 599 (353-1217) | 0.003 | 300 (226-680) | 595 (344-901) | 0.048 | 422 (192-500) | 770 (361-2396) | 0.017 |
| Faecal calprotectin > 500 μg/g, % | 21.9 | 56.4 | 0.001 | 28.6 | 53.8 | 0.038 | 9.1 | 62.1 | 0.03 |
| CRP (mg/dL) | 0.9 (0.4-2.9) | 1.9 (0.5-5.5) | 0.075 | 1.7 (0.6-7.0) | 2.4 (1.0-6.4) | 0.327 | 0.18 (0.1-0.7) | 0.5 (0.2-3.1) | 0.028 |
| ESR (mm/h) | 25 (12-48) | 35 (21-56) | 0.079 | 32 (13-57) | 37 (24-62) | 0.134 | 18 (8-39) | 25 (9-45) | 0.480 |
| Hb (g/dl) | 12 (10.4-13.0) | 11.7 (10.3-12.2) | 0.262 | 11.6 (10.2-12.2) | 11.5 (10.6-12.1) | 0.935 | 13 (10.5-14.2) | 11.7 (9.7-13.0) | 0.119 |
| Htc (%) | 37 (32-40) | 35 (32-38) | 0.045 | 37.3 (32.8-38.7) | 25.1 (33.0-37.0) | 0.123 | 40 (31-42) | 34 (29-40) | 0.170 |
| Platelets (x10^9^/L) | 400 (330-511) | 472 (369-560) | 0.195 | 451 (359-627) | 479 (397-567) | 0.549 | 366 (321-409) | 385 (290-553) | 0.435 |
| Orosomucoid (mg/L) | 118 (83-177) | 252 (141-308) | 0.028 | 228 (228-228) | 252 (141-308) | 0.909 | 105 (78-124) | - | - |
| Prolonged Interval was defined as an overall time greater than P75. Interval 3: Total: 34 days; CD: 49 days; UC: 29 days. PI: prolonged interval. nPI: no prolonged interval. CD: Crohn’s disease. UC: Ulcerative colitis. CRP: C-reactive protein; Hb: haemoglobin; Hct: haematocrit; ESR: Erythrocyte sedimentation rate; EIM: extraintestinal manifestations; IQR: Interquartile range. The 4 patients with IBDU were excluded from the analysis | | | | | | | | | |

| **Table S5. Interval 2. Hypothesis contrast** | | | | | | | | | |
| --- | --- | --- | --- | --- | --- | --- | --- | --- | --- |
|  | **All** | | | **CD** | | | **UC** | | |
| **Variable** | **DD** | **nDD** | **p** | **DD** | **nDD** | **p** | **DD** | **nDD** | **p** |
| Gender (Female), % | 36.1 | 42.2 | 0.329 | 37.5 | 31.5 | 0.380 | 33.3 | 63.9 | 0.066 |
| Family history, % | 13.9 | 19.4 | 0.316 | 12.5 | 21.9 | 0.244 | 16.7 | 14.3 | 0.583 |
| Rural zone, % | 8.3 | 13.8 | 0.296 | 4.2 | 13.7 | 0.186 | 16.7 | 13.9 | 0.569 |
| MDO > P50* | 55.6 | 41.3 | 0.097 | 58.3 | 38.4 | 0.070 | 50.0 | 47.2 | 0.565 |
| MDO > P75** | 36.1 | 14.7 | 0.007 | 41.7 | 16.4 | 0.014 | 25.0 | 11.1 | 0.231 |
| MDO, median, IQR | 4 (2-9) | 3 (1-5) | 0.012 | 7 (2-13) | 3 (1-6) | 0.007 | 2 (1-5) | 2 (1-4) | 0.397 |
| Referral from Primary Care | 60.7 | 46.2 | 0.129 | 56.3 | 48.4 | 0.390 | 66.7 | 41.4 | 0.129 |
| Referral during hospitalization | 16.7 | 35.2 | 0.027 | 16.7 | 35.6 | 0.065 | 16.7 | 34.3 | 0.220 |
| Diarrhea, % | 33.3 | 38.5 | 0.362 | 29.2 | 45.2 | 0.125 | 41.7 | 25.0 | 0.228 |
| Abdominal pain, % | 30.6 | 32.1 | 0.518 | 37.5 | 38.4 | 0.570 | 16.7 | 19.4 | 0.601 |
| Rectal bleeding, % | 13.9 | 20.2 | 0.283 | 4.2 | 5.5 | 0.638 | 33.3 | 50.0 | 0.253 |
| Perianal disease, % | - | - | - | 16.7 | 4.1 | 0.061 | - | - | - |
| EIM, % | 11.8 | 17.6 | 0.304 | 9.1 | 20.5 | 0.183 | 16.7 | 11.4 | 0.486 |
| Severe disease (wPCDAI/PUCAI),% | 18.2 | 32.7 | 0.081 | 17.4 | 41.1 | 0.030 | 20.0 | 14.7 | 0.509 |
| Z score weight, median IRQ | -0.31 (-1.03-0.75) | -0.48 (-0.92-0.33) | 0.930 | -0.36 (-1.13- -0.12) | -0.53 (-0.95-0.06) | 0.755 | 0.14 (-0.9-1.4) | -0.16 (-0.91-0.67) | 0.505 |
| Z score height, median IQR | 0.11 (-0.81-0.82) | -0.11 (-0.8-0.84) | 0.713 | -0.17 (-1.2-0.59) | -0.14 (-0.83-0.76) | 0.827 | 0.67 (-0.26-1.38) | 0.13 (-0.5-1.2) | 0.372 |
| Faecal calprotectin (μg/g) | 413 (246-785) | 507 (309-1060) | 0.124 | 585 (257-967) | 500 (300-871) | 0.900 | 369 (158-491) | 770 (437-2100) | 0.005 |
| Faecal calprotectin > 500 μg/g, % | 38.7 | 51.0 | 0.162 | 52.4 | 46.2 | 0.403 | 10.0 | 61.3 | 0.006 |
| CRP (mg/dL) | 0.81 (0.3-1.8) | 2.15 (0.56-6.09) | 0.008 | 1.14 (0.8-3.5) | 2.8 (1.2-7.3) | 0.049 | 0.18 (0.12-0.5) | 0.58 (0.22-3.18) | 0.012 |
| CRP > 3 mg/dl | 67.6 | 77.5 | 0.179 | 91.3 | 91.0 | 0.668 | 18.2 | 51.4 | 0.053 |
| ESR (mm/h) | 26 (11-41) | 35 (20-60) | 0.023 | 26 (15-47) | 40 (22-67) | 0.021 | 23 (6-39) | 24 (9-47) | 0.396 |
| Hb (g/dl) | 11.5 (10.3-12.9) | 11.8 (10.2-12.2) | 0.570 | 11.3 (10.3-12.2) | 11.7 (10.6-12.1) | 0.795 | 13.3 (8.3-14.2) | 11.6 (9.7-12.9) | 0.268 |
| Htc (%) | 35.6 (32.7-39.5) | 35.1 (32.2-38.3) | 0.619 | 35.2 (33.3-37.0) | 35.7 (33-38.3) | 0.781 | 40.8 (26.8-42.5) | 34.3 (30.3-38.9) | 0.408 |
| Platelets (x10^9^/L) | 412 (314-532) | 460 (370-560) | 0.274 | 469 (371-584) | 478 (389-570) | 0.759 | 333 (286-405) | 387 (316-487) | 0.225 |
| Orosomucoid (mg/L) | 110 (93-110) | 228 (123-289) | 0.229 | - | - | - | 110 (93-110) | 95 (73-95) | 0.667 |
| Orosomucoid > 150 mg/L | 0 | 61.5 | 0.200 | - | - | - | - | - | - |
| Prolonged Interval was defined as an overall time greater than P75. Interval 5: Total: 8.7 months; CD: 9.8 months; UC: 4.9 months PI: prolonged interval. nPI: no prolonged interval. CD: Crohn’s disease. UC: Ulcerative colitis. CRP: C-reactive protein; Hb: haemoglobin; Hct: haematocrit; ESR: Erythrocyte sedimentation rate; EIM: extraintestinal manifestations; IQR: Interquartile range. The 4 patients with IBDU were excluded from the analysis | | | | | | | | | |
